# The utero-tonic effects of low dose intravenous ketamine in cesarean section under spinal anesthesia; A randomized double-blind clinical trial

**DOI:** 10.22088/cjim.14.2.218

**Published:** 2023

**Authors:** Mohammad Haghighi, Soheil Soltanipour, Farnoush Farzi, Mandana Mansour Ghanaie, Gelareh Biazar, Azadeh Malekzadeh, Mahin Tayefeh Ashrafiyeh

**Affiliations:** 1 Anesthesiology Research Center, Department of Anesthesiology, Alzahra hospital, Guilan University of Medical Sciences , Rasht, Iran; 2 Department of Community Medicine, School of Medicine, Guilan University of Medical Sciences, Rasht, Iran; 3 Reproductive Health Research Center, Department of Obstetrics and Gynecology, Al-Zahra Hospital, School of Medicine, Guilan University of Medical Sciences, Rasht, Iran

**Keywords:** Ketamine, Cesarean Section, Spinal Anesthesia

## Abstract

**Background:**

Recently, the prevalence of cesarean section (CS) has been on the rise and the proper uterine tone is an important issue. We investigated the effects of intravenous (IV) ketamine on intraoperative bleeding and the need for oxytocin in CS under spinal anesthesia (SA).

**Methods:**

This study, took place at Alzahra hospital during 2020. Pregnant women candidate for elective CS under SA were divided into two groups of ketamine and placebo. In group K, after umbilical cord clamping, 0.25 mg/kg ketamine and in group P 2ccs normal saline was injected. Mean arterial pressure and heart rate were recorded at baseline, before and 5 minutes after cord clamping and at the end of the surgery. The drop in hemoglobin values, the administrated units of oxytocin and side effects were also recorded.

**Results:**

No significant difference was found in terms of patients’ demographic data (P ≥ 0.05). The mean units of administrated oxytocin in group K was 34.61±6.63 and in group P; 48.47±12.15, which was significantly different (P=0.0001). The drop in Hb was less in group K, however not statistically significant (P=0.094). The need for methergine was significantly higher in group P (P=0.0001). The mean HR was significantly higher in group P (P=0.027), however, no significant difference was observed regarding the MAP (P=0.064). The incidence of hallucination (4.8%) and nystagmus (21%) was significantly higher in group K (P= 0.0001), but nausea and vomiting were more significant in group P (P= 0.027).

**Conclusion:**

Prophylactic administration of low-dose ketamine in CS under S.A could significantly reduce the administrated oxytocin units and the need for additional utero-tonics and was associated with less drop in Hb values.

Recently, the prevalence of cesarean section (CS) has been increasing as the most common major obstetric surgery worldwide (1). Considering several advantages of spinal anesthesia (SA) including; avoiding anesthesia-related neurotoxicity, easy to perform, fast onset of action, at least failure rate, and cost-effectiveness, it has been known as the choice of anesthesia and the preferred method (2-4). Uterine atony is the leading cause of postpartum hemorrhage (PPH) in 50- 80% of cases (5, 6). Indeed, failure of adequate uterine contraction has been known as the most common cause of PPH. While studies have demonstrated that 54- 93% of maternal mortality due to PPH can be preventable (7, 8). According to the World Health Organization (WHO) guidelines, among multiple utero tonic agents, oxytocin is recommended as the first-line drug (9). 

However, till now, a definite and clear strategy has not been available on the optimal timing, doses, routes, and regimes for the administration of this drug (10-12). Studies have reported different 50% effective dose (ED50) of oxytocin according to the mothers’ conditions such as prior history of CS (12, 13). Moreover, despite being the first choice agent, studies have reported several adverse effects related to this drug, such as headache, flushing, nausea vomiting, hypotension, tachycardia, chest pain and myocardial ischemia (14, 15). Recent studies have emphasized that small doses are associated with fewer adverse effects (16). Hence great attempts should be made to prescribe strategies to administrate the lower doses of this drug, while resulting in less blood loss as well as fewer drug-related adverse events (17). It should be noted that in the setting of CS under regional or general anesthesia, the patients are prone to the potential risk of cardio depressant agents and hemodynamic disturbances. Therefore, it is wise to investigate to find safe and effective interventions to limit the required oxytocin units and additional utero-tonic agents. In this regard, Ketamine an N-methyl-D-aspartate (NMDA) antagonist has been safely administrated in anesthetic and sub-anesthetic doses in CS, for various purposes (18). Few human and experimental studies have pointed to the utero tonic properties of ketamine, however, no comprehensive study has been planned yet (19-21). The aim of this clinical trial was to assess the effects of prophylactic low doses of IV ketamine on the amount of blood loss, the number of administrated oxytocin units, and the need for additional utero- tonic agents.

## Methods

This prospective, randomized double-blind clinical trial was conducted at Alzahra Obstetrics & Gynecology hospital in Rasht, Iran, an academic and referral center for all types of obstetric surgeries. The study protocol was approved by the institutional Ethical committee of GUMS with the ethical code of IR.GUMS.REC.1399.385 and was registered in the Iranian Registry of Clinical Trials (IRCT20170314033069N3). Moreover, informed consent was obtained from the eligible participants. 

**Inclusion criteria** ***: *** Pregnant women candidate for elective CS under SA, ASA class I, II***.***

**Exclusion criteria: **Any contraindication for Ketamine or SA, patients with coagulation disorders, changing the plan of anesthesia to general. 

**Anesthesia management and intervention: **Arriving at the operative room standard monitoring including pulse oximetry (POM), electrocardiogram (ECG), and non-invasive blood pressure (NIBP), was started. An intravenous line was fixed and an infusion of crystalloids was started. SA was performed in a sitting position at the level of L3- L4 in aseptic conditions by the needle size 26 gauge and 10 mg bupivacaine. If systolic blood pressure dropped more than 20% from baseline, 10 mg IV ephedrine was administrated. Immediately after the umbilical cord clamping, 0.25 mg/kg ketamine IV was injected in group K, and 2ccs normal saline in group P. In both groups the infusion of 20 units of oxytocin (Ampule 10mg/1cc, Caspian Company) in 1000cc isotonic serum was started. In this study similar to the study of Kaur H et al. the decision to prescribe the administration of the second-line drug, methergine was due to the lack of proper uterine contraction and inadequate response to oxytocin based on the gynecologist's assessment (22).In addition, studies have well demonstrated that following repeated doses or continuous infusion of oxytocin, desensitization of uterine oxytocin receptors occurred because of a reduction in the number of oxytocin binding sites on intact myometrial cells (23). Hemoglobin (Hb) values were checked twice; at baseline and 4 hours post-operation and the drop in the Hb values was calculated. Hemodynamic parameters including mean arterial pressure (MAP), and heart rate (HR) were recorded, at baseline, before cord clamping, 5 minutes after clamping, and at the end of the surgery. The number of administrated units of oxytocin and the adverse events were also recorded in two groups.   

**Randomization & Blinding: **Eligible patients were divided into two groups of ketamine and placebo by using a list created by computer and WINPEPI Ver.11.65 software in the form of 4 blocks prepared by the project methodology consultant. These patients were assigned into two groups of Ketamine; group K (n=105) and Placebo; group P (n=105). Patients and the outcome assessor were blinded to the study groups. Considering the influencing items like the surgeon’s experience on intraoperative bleeding and estimation of uterine contractility all the operations were performed by a single surgeon. 

**Statistical analysis: **To analyze the data SPSS version 21 was used and Chi-square, Fisher, and t-tests were applied. The parametric data were expressed as mean ± standard deviation and nonparametric data as median (range). Presenting our results, a p-value less than 0.05 was considered as significant.

**Sample size:** According to G* Power 3.1.9.2 software, considering two tails, medium effect size of 0.5, α and (1-β) errors 0.05 and 0.95 respectively, and allocation ratio 1/1, 105 people in each group were considered as a sufficient sample size.

## Results

**Figure 1:**
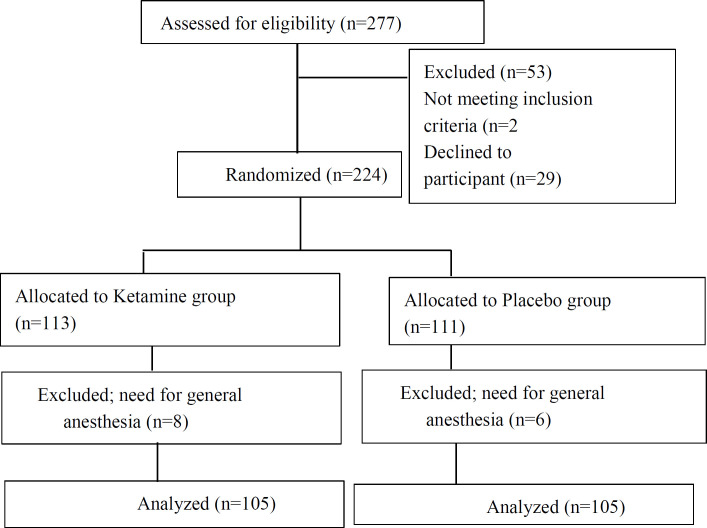
CONSORT flow diagram of the survey

During the study period, 277 pregnant women were screened for eligibility and finally, the data from 210 were analyzed (fig 1). The mean age of the enrolled women was 30.87± 5.57 years, the mean BMI 28.29± 3.23 kg/m2, and the mean surgery duration 50.54±6.17 minutes. No significant difference was found in terms of patients’ demographic data (Table 1). The mean units of administrated oxytocin in group K was 34.61±6.63 and in group P; 48.47±12.15, which was significantly different (P=0.0001). The need for methergine was significantly higher in group P (P=0.0001). In terms of hemodynamic parameters, the mean HR was significantly higher in group P (P= 0.027), however, no significant difference was observed regarding MAP values (P=0.064) (Table 2). The amount of bleeding presented in Hb values drop was less in group K, however not statistically significant (P=0.094) (Table 3). Women in group K showed significantly more hallucination symptoms (4.8%) compared with group P (0%) (P= 0.024) and transient nystagmus (21%) compared with group P (0%) (P= 0.0001) respectively. The incidence of nausea and vomiting was significantly higher in group P (32.4%) compared with group K (19%) (P= 0.027) (Table 4). In order to predict the effect of independent variables, multiple linear regression analysis by Enter method was applied. 

It was found that none of the variables including; the type of groups placebo or ketamine, age, BMI, ASA class, MAP, gravidity, and parity significantly predict Hb changes (P=0.697). However in terms of the consumed oxytocin units, it was found that the types of groups (Ketamine or placebo) and age were significant predictors (F changes P <0.001) (Watson=Durbin-1.99) (Table 5).

**Table 1:** Comparing maternal and neonatal demographic data

Variables		Ketamine		Placebo		P value
		Number	Percent	Number	Percent	
Age (Year)	Less than 30	46	43.8	56	53.3	0.167
	More than 30	59	56.2	49	46.7	
Age (Year) Mean ± SD (min-max)		31.49±5.88		30.25±5.2		0.109
BMI (kg/m2)	Less than 19	0	0	0	0	0.317
	19-25	20	19	18	17.1	
	25-30	59	56.2	51	48.6	
	More than 30	26	24.8	36	34.3	
BMI (kg/m2)Mean ± SD		28 ± 3.07		28.59±3.38		0.187
ASA Class	I	89	84.8	81	77.1	0.16
	II	16	15.2	24	22.9	
Gravidity	1	29	27.6	37	35.2	0.285
	2	45	42.9	46	43.8	
	3 and more	31	29.5	22	21	
Gravidity (Mean ± SD )		2.06±0.85		1.85±0.73		0.059
Parity	0	31	29.5	37	35.2	0.437
	1	55	52.4	55	52.4	
	2 and more	19	18.1	13	12.4	
Parity (Mean ± SD )		0.89 ± 0.7		0.77±0.65		0.189
Gestational age (Mean ± SD )		37.68±1.07		37.8±1.22		0.438
Birth weight (g) (Mean ± SD )		3109.8±384		3207.8±379		0.064

**Table 2:** Comparing the outcomes between the two groups of ketamine & placebo

Outcome	Ketamine	Placebo	P value	Effect Size	Confidence Interval (95%)
Duration of surgery (Minutes) (Mean ± SD)	50.23±5.15	50.85±7.05	0.469	Cohen's d= - 0.1004	((- 0.37)-0.17)
Received oxytocin (unit) (Mean ± SD)	36.61±6.63	48.47±12.15	0.0001	Cohen's d= - 1.21	**(**(- 1.5)-(- 0.91)**)**
Women who needed methergine (number)	9(8.6%)	41(39%)	0.0001	RR = 0.14	(0.06-0.28)
Mean arterial pressure (mmHg) (Mean ± SD)	9.84±87.62	85.17±9.17	0.064	Cohen's d= 0.25	((-0.016)- 0.52)
Heart rate (minute) (Mean ± SD)	87.6± 9.84	88.74±12.92	0.027	Cohen's d= - 0.30	**(**- 0.51-(- 0.58)**)**

**Table 3:** Comparing of the drop in Hb values between the two groups

Group	Before surgery (Mean ± SD)	After surgery (Mean ± SD)	In-group statistical estimation	Differences in hemoglobin before and after surgery (Mean ± SD)	P value
Ketamine	11.8±1.32	10.58±1.33	P=0.0001	1.22±0.36	0.094
Placebo	11.81±0.96	10.5±1.01	P=0.0001	1.3±0.36	
Statistical estimation in each time period	P=0.952	P=0.651			

**Table 4:** Comparison of the frequency of complications between the two groups

Side effects	Status	Ketamine		Placebo		Total		P value
		Number	Percent	Number	Percent	Number	Percent	
Nausea & vomiting	Yes	20	19	34	32.4	54	25.7	0.027
	No	85	81	71	67.6	156	74.3	
Hypotension	Yes	26	24.8	35	33.3	61	29	0.171
	No	79	75.2	70	66.7	149	71	
Hallucination in recovery	Yes	5	4.8	0	0	5	2.4	0.024
	No	100	95.2	105	100	205	97.6	
Nystagmus	Yes	22	21	0	0	22	10.5	0.0001
	No	83	79	105	100	188	89.5	

**Table 5:** Multiple Linear Regression Analysis to Predict Oxytocin Consumption

	**B**	**SEB**	**β**	**P** **value**
Constant	25.54	8.79		0.004
Intervention group )Ketamine compared to placebo(	-12.27	1.36	-0.53	<0.001
Age	0.43	0.15	0.21	0.005
BMI	0.14	0.21	0.04	0.494
ASA Class	0.13	1.92	0.005	0.942
Mean arterial pressure(MAP)	0.11	0.07	0.09	0.115
Gravidity	-3.67	2.09	-0.26	0.074
Parity	3.99	2.36	0.23	0.092

(R²=0.331)

## Discussion

This study, for the first time, investigated the effects of low-dose IV ketamine on the amount of bleeding and oxytocin consumption in CS under SA. According to the results of this study, low-dose IV ketamine showed beneficial clinical effects in CS as the need for additional utero-tonic agents and administrated oxytocin units were significantly lower in group K. The drop in Hb values was also less in group K. The utero-tonic effects of ketamine have been investigated by a limited number of studies and the majority of them were experimental. Oats J et al. for the first time focused on the issue, noting that attention towards the utero tonic effects of ketamine was drawn when gynecologists reported less intra-post-operative bleeding in the pregnant uterus (19). 

However the practical and potential utero-tonics properties of ketamine remained silent for many years, and so far no well-planned clinical trial has been designed in this area. Although the lack of relevant studies indicates the novelty of this work, comparing the results of studies and a challenging discussion on the similarities and differences would be impossible. In addition to the lack of similar studies, the real underlying mechanism of utero- tonic effects of ketamine has not been well explored. Gallon et al. used ketamine for dilation and curettage surgery and observed promising clinical findings. The uterus was markedly small and contracted at the end of the surgery. Blood loss was also minimal intra and post-operative. It was noticeable that there was no need for oxytocin administration because the uterus was sufficiently contracted and firm (24). Oats J. N et al. demonstrated that IV ketamine had uterine contraction properties similar to ergometrine (19). Korie O et al. found that low doses of oral ketamine in term uterus had oxytocin-like effects. However, they stated that this result should be confirmed by further trials and the underlying responsible mechanism must be clarified (25). 

Moreover, as another hypothetical mechanism, studies have shown that serum noradrenaline levels increase following the administration of ketamine, which can cause uterine contractions (26). In this study, no serious side effects were reported. Women in group K were less affected by nausea & vomiting which supported a number of previous studies, discussing the responsible underlying mechanism. Ketamine is an anesthetic agent with N-methyl-Daspartate (NMDA)-antagonistic properties. Sub-anesthetic doses of ketamine inhibit development of acute opioid tolerance, hyperalgesia, and central sensitization. Considering that visceral pain and vagal stimulation, sympathetic block and hypotension may be the main mechanisms involved in developing nausea & vomiting in SA,  ketamine can prevent the incidence of nausea & vomiting by its unique central sympathomimetic, vagolytic, and analgesic properties (27, 28). In addition to the mentioned mechanisms, since nausea & vomiting are some of the adverse effects of oxytocin, and due to the fact that the administrated units of oxytocin were significantly less in group k, less probability of nausea & vomiting was expected in the intervention group (29). Shabana A et al. showed that the use of IV ketamine in parturient subjected to CS under SA was associated with a significant reduction in the incidence of intra-operative nausea & vomiting and hypotensive episodes (30). Furthermore, studies have indicated several advantages of IV ketamine in CS. Wang J et al. in a meta-analysis including 20 RCTs with 1.737 women underwent CS, found that intraoperative IV ketamine could significantly reduce postoperative pain score and the amount of morphine consumption (31). Jiaxin Y et al. reported that IV ketamine (0.25 mg/kg) during CS could reduce the symptoms of postpartum depression (32). Zangouei A et al. reported that low-dose intravenous ketamine could significantly prevent post-SA headache in CS (33). Reduction of post-SA shivering in CS(34), and maintaining stable hemodynamic status (35) have been considered as the other advantages of IV ketamine in CS. In general, the current evidence on the subject is so limited, and considering several other clinical benefits of ketamine as a safe, available, and cost-effective agent, it is worth planning for further studies in the future. Despite the valuable practical findings of this study, we acknowledge some limitations; first, it was a single-center study. Second, women candidates for elective CS under SA enrolled in the survey and those who underwent general anesthesia (GA) were excluded. While, in GA, several anesthetic agents such as volatile anesthetics with muscle relaxant properties are used. Therefore, the problem could be more important in GA cases. In addition, emergency cases and higher ASA classes were excluded which could be a pitfall of this study (22).Third, Hb values might not be the optimal reliable marker of blood loss as it is affected by the mothers' hydration status. Especially with the i.v. fluids loading which is administrated for SA, and physiologic changes in maternal blood volume (36). Fourth, insufficient uterine tone and the need for more utero- tonic agents were assessed by the obstetrician which could be influenced by subjective estimation. However, in order to limit this factor all the operations were performed by a single and experienced surgeon. Prophylactic administration of low-dose IV ketamine in CS under SA could significantly reduce the required doses of oxytocin and the need for other utero-tonics and also the drop in Hb levels. Further researches are welcomed to find practical and generalizable results in a clinical setting.

## Funding:

This research received no financial support, for preparing or publication of this paper.

## Conflicts of Interest:

None declared.

## Authors’ contribution:

M.H conceptualized and designed the study, G.B wrote the first draft of the manuscript; G.B, M.T, MMG & F.F collaborated in the data collection process. S.S: performed statistical analysis; collaborated in data processing, interpreted the results; G.B: edited and critically reviewed manuscript; All authors reviewed and approved the final draft of the manuscript.
